# Trajectories of a Composite Lipid, Glucose, and Refined Obesity Index Predict Cardiovascular Disease

**DOI:** 10.1155/cdr/1092234

**Published:** 2026-11-04

**Authors:** Linying Xia, Tengfei Ji, Yuan Wang, Danni Li, Yuyu Sun, Wenwen Wang, Jie Deng

**Affiliations:** ^1^ Department of Cardiology The Second Affiliated Hospital of Xi′an Jiaotong University Xi′an Shaanxi China; ^2^ Shaanxi International Travel Healthcare Center (Xi′an Customs Port Outpatient Department) Xi′an Shaanxi China; ^3^ Department of Epidemiology Ministry of Education Key Lab of Hazard Assessment and Control in Special Operational Environment School of Public Health Fourth Military Medical University Xi′an Shaanxi China fmmu.edu.cn

**Keywords:** cardiovascular disease, CHG-refined obesity derivatives, cholesterol-HDL-glucose, insulin resistance, longitudinal trajectories, refined obesity derivatives

## Abstract

**Background:**

Insulin resistance and central adiposity are key drivers of cardiovascular disease (CVD). Although the CHG index and obesity‐related indicators can separately predict metabolic risks, this study explored the predictive value of their composite indices and longitudinal trajectories for incident CVD.

**Methods:**

Based on 4164 middle‐aged and elderly participants from a national prospective cohort, we analyzed baseline levels, cumulative exposures and K‐means‐defined longitudinal trajectories of CHG‐related composite indices. Cox regression, ROC analysis, and weighted quantile sum regression were adopted to explore CVD associations and predictive efficiency.

**Results:**

During an 8‐year median follow‐up, 22.14% of participants developed CVD. Elevated baseline values, increased cumulative exposure, and unfavorable trajectories of composite indices were linked to higher CVD risk. Per 1‐SD rise in baseline CHG‐WC and CHG‐CVAI corresponded to 19% and 17% higher CVD risk, respectively. Their addition produced statistically significant improvements in predictive performance and risk stratifying capacity of the Framingham Risk Score, as further supported by NRI and IDI analyses.

**Conclusion:**

Longitudinal trajectories and cumulative exposures of CHG‐obesity composite indices serve as reliable independent predictors of CVD. Dynamic monitoring of these indicators facilitates early CVD risk stratification.

## 1. Introduction

The prevalence of cardiovascular disease (CVD) continues to rise, posing a severe global public health challenge (1–3). This burden is further exacerbated by the globally aging population (4). As the leading cause of morbidity and mortality worldwide, CVD imposes a disproportionate burden on less developed regions and exhibits a conspicuous trend toward earlier onset (5). In 2023, an estimated 19.2 million deaths were attributed to CVD globally, inflicting a substantial burden on patients′ families and healthcare systems (6). In recent years, the high prevalence of obesity and unhealthy lifestyles (7) has driven a continuous increase in CVD risk through pathophysiological mechanisms, including the induction of metabolic disorders (8) and vascular endothelial dysfunction (9). Therefore, it is imperative to strengthen preventive strategies focused on metabolic regulation and to actively implement primary prevention measures for CVD.

Insulin resistance (IR) is a key metabolic factor implicated in the pathogenesis of CVD (10, 11). In recent years, the triglyceride‐glucose (TyG) index has emerged as a prominent biomarker for IR, proving to be a crucial indicator for predicting the risk and prognosis of various metabolic diseases (12–14). Since its initial proposal, the cholesterol‐high‐density lipoprotein‐glucose (CHG) index (15) has demonstrated superior predictive efficacy for Type 2 diabetes compared with the TyG index. Subsequently, it has been validated to hold crucial clinical significance for CVD risk assessment in middle‐aged and older Chinese adults (16). Building on this validation, subsequent comparative studies evaluating their ability to predict CVD incidence further confirmed that CHG and TyG exhibit comparable predictive performance (17). Notably, combining IR surrogate markers with obesity derivatives yields superior predictive accuracy to that of an IR surrogate alone (18). However, existing research has primarily focused on combining TyG with traditional obesity indices, particularly body mass index (BMI). With the recent introduction of various refined obesity indices, the limitations of traditional obesity indices have become increasingly apparent. When predicting CVD risk and adverse outcomes, BMI often falls into the “obesity paradox” because of its inability to differentiate fat distribution and metabolic profiles. In contrast, refined obesity indices that incorporate dimensions such as waist circumference (WC) and lipid profiles effectively overcome the limitations of BMI in characterizing central adiposity. These precise measurement tools resolve the cognitive dilemma posed by the obesity paradox, demonstrating a stronger linear correlation with cardiovascular and metabolic health outcomes (19, 20). Wang et al. systematically investigated the association between incident CVD events and the trajectory patterns of a composite TyG‐obesity index (21). To the best of our knowledge, the associations of the baseline levels and trajectory patterns of the composite index of CHG and refined obesity derivatives (CHG‐ROD index) with the incidence of CVD remain to be elucidated. To address this knowledge gap, the present study utilized data from a large prospective cohort, the China health and retirement longitudinal study (CHARLS), to systematically evaluate the associations of both baseline levels and longitudinal trajectories of the CHG‐ROD index with incident CVD. Furthermore, we compared their predictive performance to ultimately facilitate more precise CVD risk stratification.

## 2. Methods

The present study drew upon data from the CHARLS (22). CHARLS is an ongoing, nationally representative longitudinal study designed to collect high‐quality microdata from households and individuals aged 45 years and older in China. Its primary objective is to analyze issues pertaining to population aging and to foster interdisciplinary research. The dataset covers a comprehensive range of domains, including individual and community‐level demographics, family structure, health status and anthropometric measurements, healthcare utilization, as well as socioeconomic indicators such as employment, income, wealth, and pensions. Initiated between 2011 and 2012, the baseline survey covered 450 village‐level units across 28 provinces in China, enrolling a baseline cohort of approximately 17,705 participants. Subsequent follow‐up surveys have been conducted biennially or triennially—with five waves completed to date (2011–2012, 2013, 2015, 2018, and 2020)—allowing for the continuous longitudinal tracking of health trajectories among the middle‐aged and elderly populations.

Participants were excluded if they met any of the following criteria: (1) aged under 45 years; (2) missing data on the CHG index, height, weight, or WC; (3) missing data on key covariates; or (4) loss to follow‐up. Ultimately, a total of 4164 CHARLS participants met the eligibility criteria and were included in our longitudinal analysis (Figure 1). Baseline was defined as the 2011 CHARLS survey. The exposure trajectory window spanned 2011–2015, using fasting blood and anthropometric data collected in these two waves to construct CHG‐ROD trajectories. Follow‐up commenced after the 2015 survey for the cumulative exposure analysis, whereas follow‐up for the baseline exposure analysis began after the 2011 survey. Outcome adjudication was completed by the 2020 wave, marking the end of the study.

**Figure 1:**
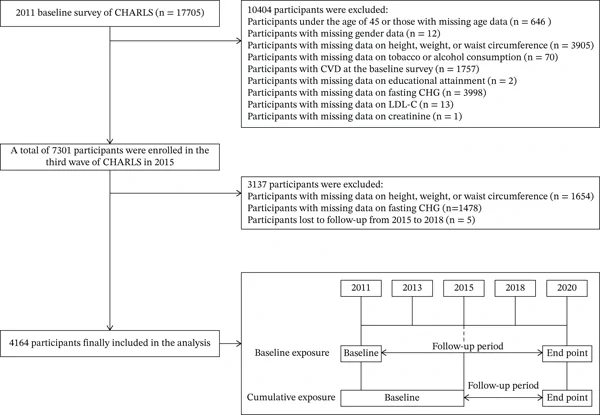
Flowchart of study population inclusion and exclusion screening based on CHARLS data. CHARLS, China Health and Retirement Longitudinal Study; CVD, cardiovascular disease; LDL‐C, low‐density lipoprotein cholesterol; CHG, cholesterol‐high‐density lipoprotein‐glucose; NON‐CVD, noncardiovascular disease.

All participants provided written informed consent, and the CHARLS protocol was approved by the Institutional Review Board of Peking University (Approval Number: IRB00001052‐11015). This study strictly adhered to the ethical principles outlined in the Declaration of Helsinki, and the reporting of results complies with the strengthening the reporting of observational studies in epidemiology guidelines.

Laboratory testing was conducted at the clinical laboratory center of capital medical university. The analyzed samples were derived from frozen plasma or whole blood, and measurements were performed utilizing an enzymatic colorimetric assay. Notably, the coefficients of variation for triglycerides (TG), total cholesterol (TC), high‐density lipoprotein cholesterol (HDL), and fasting blood glucose (FBG) were all less than 2%, indicating a high level of reliability and consistency in the measurements. The exposure variable in this study was defined as the change in the CHG index between 2012 and 2015. The formula for calculating the CHG index is as follows:

CHG index=LnTCmg/dL×FBGmg/dL2×HDLmg/dL.

Anthropometric measurements were conducted by the CHARLS research team following a unified, standardized protocol: height and weight were measured with participants wearing light clothing, whereas WC was recorded at the umbilical level at the end of a normal expiration. Based on these anthropometric parameters, several obesity indices were calculated, including BMI, waist‐to‐height ratio (WHtR), visceral adiposity index (VAI), and CVD‐associated adiposity index (CVAI). The calculations for refined obesity indices were performed as follows:

BMI=WeightkgHeightm2,

WHtR=WCcmHeightcm,

Male:VAI=WCcm39.681.88+×BMIkg/m2×TGmmol/L1.03×1.31HDLmmol/L,

Female:VAI=WCcm36.581.89+×BMIkg/m2×TGmmol/L0.81×1.52HDLmmol/L,

Male:CVAI=−267.930.680.034+×Agey+×BMIkgm2+×WCcm+2216.32×log10TGmmolL−×HDLmmolL,

Female:CVAI=−187.321.714.231.12+×Agey+×BMIkgm2+×WCcm+39.7611.66×log10TGmmolL−×HDLmmolL.

CHG–ROD is defined as CHG–ROD = CHG^∗^ ROD.

The primary outcome of our study was incident CVD. CVD was defined as a composite of heart disease and stroke. Diagnoses of heart disease and stroke were ascertained using standardized questionnaires validated in previous studies. Participants were asked: “Have you ever been told by a doctor that you have had a heart attack, coronary heart disease, angina, congestive heart failure, or other heart problems?” Stroke was assessed based on the question: “Have you ever been told by a doctor that you have had a stroke?” For the baseline exposure analysis, participants with prevalent CVD before the 2011 survey were excluded. For the cumulative exposure analysis, participants who experienced a CVD event during follow‐up prior to 2015 were censored at the time of the event and had no further follow‐up. For those without prior CVD events, follow‐up continued until the 2020 wave. The time to incident CVD was defined as follows: for baseline exposure analysis, follow‐up time was calculated from the 2011 baseline survey to the wave when the CVD event was reported; for cumulative exposure analysis, follow‐up time was calculated from the 2015 baseline survey to the wave when the CVD event was recorded.

### 2.1. Exposure Assessment

The exposure variables comprised the baseline levels of the composite CHG‐ROD indices, alongside their cumulative (cu) exposures and trajectory patterns, derived by integrating data from the baseline and third‐wave surveys. Details are provided in File S1. The following methodologies were employed to assess these exposure variables:

 cuCHG−ROD index= CHG−ROD index 20122015+CHG−ROD index 2×duration20152012−.

This study utilized the K‐means clustering algorithm to identify the trajectory patterns of the composite CHG‐ROD index from 2012 to 2015. As an unsupervised machine learning method, K‐means clustering offers the advantages of computational efficiency and visualization capabilities. The optimal number of clusters was jointly determined using the elbow method and silhouette coefficient. We tested *k* from 2 to 6 for each composite index. The elbow plot showed (Figure 2) that the within‐cluster sum of squares declined markedly at *k* = 2 and plateaued with larger *k*. When *k* = 2, the silhouette coefficient was calculated (Table S1). All silhouette coefficients are greater than 0.5, indicating a good clustering structure. Higher *k* generated small, clinically uninterpretable subgroups. Thus, *k* = 2 was selected, yielding “well‐controlled” and “poorly controlled” trajectory groups. Each trajectory was constructed using two repeated measurements (2011 and 2015).

**Figure 2:**
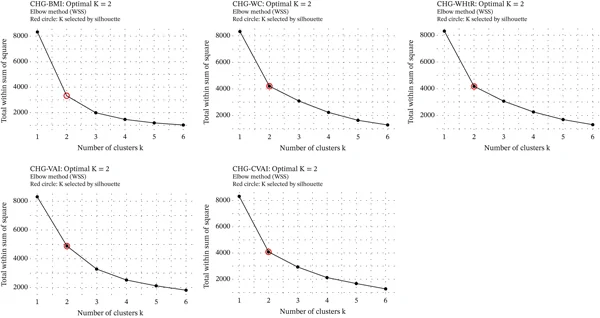
Determination of the optimal number of clusters for CHG‐ROD trajectories. The optimal number of clusters (K) for the longitudinal trajectories of the CHG‐ROD was determined using the elbow method (WSS). Two clusters were identified via the K‐means method (based on Euclidean distance) for CHG‐BMI, CHG‐WC, CHG‐WHtR, CHG‐VAI, and CHG‐CVAI. CHG, cholesterol‐high‐density lipoprotein‐glucose; CHG‐ROD, the composite index of CHG and refined obesity derivatives; WSS, Within‐Cluster Sum of Square; WC, waist circumference; WHtR, waist‐to‐height ratio; BMI, body mass index; VAI, visceral adiposity index; CVAI, CVD‐associated adiposity index.

Ultimately, trajectory patterns of the CHG‐ROD were identified (Figures 3 and 4). Based on clustering analysis, all composite indices (CHG‐BMI, CHG‐WC, CHG‐WHtR, CHG‐VAI, and CHG‐CVAI) were categorized into two distinct trajectories. CHG‐BMI, CHG‐WC, CHG‐WHtR ,and CHG‐VAI exhibited stable trends in both trajectories: Pattern 1 (well‐controlled) with persistently low levels and Pattern 2 (poorly controlled) with persistently high levels. In contrast, CHG‐CVAI exhibited a slight upward trend from 2012 to 2015 in both trajectory groups, which differed from the stable patterns of the other four indices. Nevertheless, this mild increase did not affect their fundamental distinction: the poorly controlled group remained at unfavorable levels, whereas the well‐controlled group maintained favorable status throughout the study period. With two time points, clustering separates individuals by their average index level and the change between surveys, rather than complex curved longitudinal patterns.

**Figure 3:**
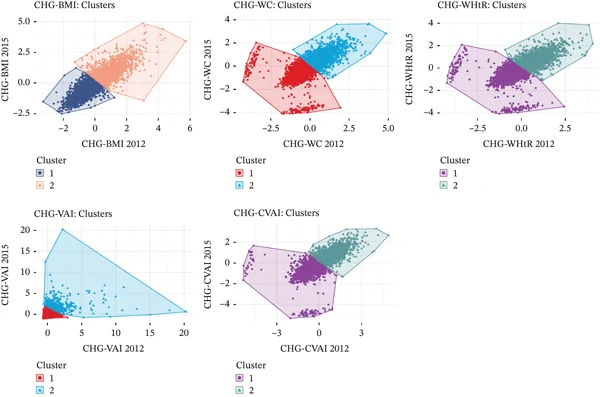
K‐means trajectory patterns: CHG‐ROD scatter plots 2012 versus 2015. A scatter plot comparing standardized values of CHG‐BMI, CHG‐WC, CHG‐WHtR, CHG‐VAI, and CHG‐CVAI between 2012 (*x*‐axis) and 2015 (*y*‐axis). Each cluster is marked by a unique color and shape to distinguish groups and highlight temporal variations, with two‐cluster patterns identified for CHG‐BMI, CHG‐WC, CHG‐WHtR, CHG‐VAI, and CHG‐CVAI. Clustering analysis categorized these composite indices into two trajectories: Pattern 1 (well‐controlled) and Pattern 2 (poorly controlled). CHG, cholesterol‐high‐density lipoprotein‐glucose; CHG‐ROD, the composite index of CHG and refined obesity derivatives; WC, waist circumference; WHtR, waist‐to‐height ratio; BMI, body mass index; VAI, visceral adiposity index; CVAI, CVD‐associated adiposity index.

**Figure 4:**
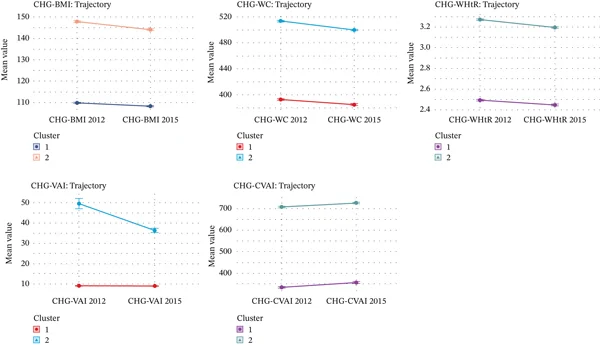
K‐means trajectory patterns: CHG‐ROD line plots 2012–2015. Line graphs display mean values with error bars for each cluster (2012–2015), illustrating dynamic changes in metabolic‐adiposity markers over the study period. These graphs highlight two distinct profiles: consistently well‐controlled and poorly controlled. CHG‐BMI, CHG‐WC, CHG‐WHtR, and CHG‐VAI maintained stable trajectories (persistently low or high levels), whereas CHG‐CVAI showed a slight upward trend in both clusters from 2012 to 2015, differing from the other four indices. CHG, cholesterol‐high‐density lipoprotein‐glucose; CHG‐ROD, the composite index of CHG and refined obesity derivatives; WC, waist circumference; WHtR, waist‐to‐height ratio; BMI, body mass index; VAI: visceral adiposity index; CVAI, CVD‐associated adiposity index.

### 2.2. Statistical Analysis

Participants were stratified based on the incidence of CVD. Continuous variables with a normal distribution were presented as mean ± standard deviation (SD), and intergroup comparisons were performed using the independent samples *t*‐test. Continuous variables with a skewed distribution were expressed as medians with interquartile ranges (IQRs), and compared using the Mann–Whitney *U* test. Categorical variables were presented as frequencies and percentages (*n*, %), with intergroup differences evaluated using the chi‐square test or Fisher′s exact test when the expected frequency in any cell was less than five. With two time points, clustering separates individuals by their average index level and the change between surveys, rather than complex curved longitudinal patterns.

As previously described, the K‐means clustering algorithm was applied to the composite CHG‐ROD index, measured in 2012 and 2015, to identify distinct trajectory patterns. To evaluate the association of CHG‐ROD with the incidence of the primary outcome events, we categorized CHG‐ROD into four groups according to quartiles (Q1–Q4) of baseline levels and cumulative levels, respectively. Meanwhile, K‐means clustering trajectories clustering was applied to identify trajectory patterns of CHG‐ROD. Kaplan–Meier analysis was performed for the above three grouping strategies, and the log‐rank test was used to compare differences in the incidence of primary outcome events among groups.

Cox proportional hazards regression models were constructed to examine the associations of baseline quantile, trajectory cluster, and cumulative quantile of these indices with the risk of incident CVD, with four sequential models applied for adjustment. Model 1 was unadjusted; Model 2 was adjusted for age and gender; Model 3 was further adjusted for drinking status, smoking status, hypertension, diabetes, marital status, education, and dyslipidemia based on Model 2. Model 4 was additionally adjusted for creatinine (Cr) and high‐sensitivity C‐reactive protein (hs‐CRP) based on Model 3. Variance inflation factors (VIFs) were calculated in Model 4 to assess multicollinearity, with a VIF exceeding 10 indicating high multicollinearity. No significant multicollinearity was observed in our study (Table S2).

Furthermore, restricted cubic spline (RCS) regression analysis was employed to explore the potential nonlinear relationships between these indices and incident CVD. To evaluate the predictive performance of the baseline and cumulative exposures for CVD, receiver operating characteristic (ROC) curve analysis was conducted. To further confirm its clinically relevant predictive advantage, the composite CHG‐ROD index was evaluated against the CHG index alone via DeLong′s test. Additionally, we investigated the incremental predictive value of adding these indices to the Framingham Risk Score (FRS) model, and pairwise comparisons were performed using the DeLong test to determine whether the addition of these indices improved predictive performance. The incremental predictive value was further assessed using the C‐index, integrated discrimination improvement (IDI), and net reclassification improvement (NRI).

Given that the composite CHG‐ROD indices are derived from routine clinical parameters, this study employed a weighted quantile sum (WQS) regression with 1000 bootstrap iterations. This approach allowed us to systematically evaluate the differential contributions of obesity indices and various metabolic components to the risk of incident CVD under long‐term exposure. This model assigns standardized empirical weights (ranging from 0 to 1), where the weight coefficient of each component directly quantifies its relative contribution to the overall CVD risk.

To assess the robustness of our findings, multiple sensitivity analyses were conducted. First, subgroup analyses stratified by age, gender, smoking status, drinking status, hypertension, diabetes, and dyslipidemia were performed to examine the associations of the baseline levels, cumulative exposures, and trajectory patterns of these indices with CVD risk across different subpopulations. All subgroup models were fully adjusted as in Model 4. Second, to mitigate potential reverse causality bias, the association analyses were repeated after excluding participants who developed CVD during the third and fourth survey waves. All statistical analyses were performed using R software (Version 4.1.2), and statistical significance was defined as a two‐sided *p* − value < 0.05.

## 3. Results

### 3.1. Baseline Characteristics

As shown in Table 1, the present study analyzed a total of 4164 individuals. Over a median follow‐up of 8 years, 922 participants developed incident CVD, accounting for 22.14% of the cohort. Participants with incident CVD were more likely to be older females who were nonsmokers and nondrinkers. They presented with higher weight, WC, and BMI, as well as a higher prevalence of comorbidities including dyslipidemia, hypertension, and diabetes. Additionally, they exhibited elevated levels of blood glucose, TC, low‐density lipoprotein cholesterol (LDL‐C), and TG, alongside lower HDL. Regarding the baseline and cumulative exposure levels of the CHG‐ROD index, participants who developed CVD during follow‐up demonstrated significantly higher values. In terms of trajectory patterns, participants with incident CVD generally exhibited poorer control status across these indices.

**Table 1:** Baseline characteristics, cumulative exposures, and longitudinal trajectories of CHG‐ROD stratified by CVD.

Variables	Total (*n* = 4164)	0 (*n* = 3242)	1 (*n* = 922)	Statistic	*p*
Age, mean ± SD	58.69 ± 8.64	58.47 ± 8.75	59.49 ± 8.20	*t* = −3.28	0.001
Height, mean ± SD	1.58 ± 0.08	1.58 ± 0.08	1.58 ± 0.08	*t* = 0.17	0.862
Weight, mean ± SD	58.74 ± 10.66	58.24 ± 10.50	60.48 ± 11.02	*t* = −5.49	< 0.001
Waist, mean ± SD	84.28 ± 12.17	83.70 ± 11.89	86.33 ± 12.92	*t* = −5.81	< 0.001
BMI, mean ± SD	23.48 ± 3.51	23.28 ± 3.44	24.20 ± 3.69	*t* = −6.76	< 0.001
Gender, *n* (%)				*χ* ^2^ = 8.95	0.003
Male	1901 (45.65)	1520 (46.88)	381 (41.32)		
Female	2263 (54.35)	1722 (53.12)	541 (58.68)		
Drinking status, *n* (%)				*χ* ^2^ = 8.28	0.004
No	2748 (65.99)	2103 (64.87)	645 (69.96)		
Yes	1416 (34.01)	1139 (35.13)	277 (30.04)		
Smoking status, *n* (%)				*χ* ^2^ = 5.74	0.017
No	2911 (69.91)	2237 (69.00)	674 (73.10)		
Yes	1253 (30.09)	1005 (31.00)	248 (26.90)		
Marital status, *n* (%)				*χ* ^2^ = 0.13	0.715
Other	447 (10.73)	345 (10.64)	102 (11.06)		
Married	3717 (89.27)	2897 (89.36)	820 (88.94)		
Education, *n* (%)				*χ* ^2^ = 1.73	0.422
Under high school	3787 (90.95)	2953 (91.09)	834 (90.46)		
High school or equivalent	271 (6.51)	212 (6.54)	59 (6.40)		
Above high school	106 (2.55)	77 (2.38)	29 (3.15)		
Hypertension, *n* (%)				*χ* ^2^ = 56.03	< 0.001
No	2589 (62.18)	2113 (65.18)	476 (51.63)		
Yes	1575 (37.82)	1129 (34.82)	446 (48.37)		
Diabetes, *n* (%)				*χ* ^2^ = 10.14	0.001
No	3555 (85.37)	2798 (86.30)	757 (82.10)		
Yes	609 (14.63)	444 (13.70)	165 (17.90)		
Dyslipidemia, *n* (%)				*χ* ^2^ = 53.50	< 0.001
No	3786 (90.92)	3004 (92.66)	782 (84.82)		
Yes	378 (9.08)	238 (7.34)	140 (15.18)		
WHtR, mean ± SD	0.53 ± 0.08	0.53 ± 0.08	0.55 ± 0.08	*t* = −5.47	< 0.001
VAI, mean ± SD	2.23 ± 2.91	2.16 ± 2.96	2.46 ± 2.72	*t* = −2.68	0.007
CVAI, mean ± SD	92.91 ± 42.85	90.22 ± 42.58	102.37 ± 42.46	*t* = −7.65	< 0.001
FPG, mean ± SD	109.13 ± 32.97	107.89 ± 30.79	113.52 ± 39.40	*t* = −4.01	< 0.001
TC, mean ± SD	194.26 ± 37.88	193.08 ± 37.62	198.39 ± 38.51	*t* = −3.76	< 0.001
HDL, mean ± SD	51.47 ± 15.21	51.94 ± 15.20	49.81 ± 15.15	*t* = 3.76	< 0.001
LDL‐C, mean ± SD	117.52 ± 34.72	116.39 ± 34.30	121.49 ± 35.90	*t* = −3.94	< 0.001
TG, mean ± SD	127.72 ± 91.10	125.45 ± 91.62	135.72 ± 88.86	*t* = −3.02	0.003
Cr, mean ± SD	0.76 ± 0.18	0.77 ± 0.18	0.76 ± 0.18	*t* = 1.43	0.154
hs‐CRP, mean ± SD	2.53 ± 6.82	2.46 ± 6.77	2.76 ± 6.99	*t* = −1.17	0.242
CHG‐BMI, mean ± SD	125.49 ± 24.09	123.82 ± 23.44	131.37 ± 25.41	*t* = −8.11	< 0.001
CHG‐WC, mean ± SD	450.04 ± 82.99	444.84 ± 80.69	468.35 ± 88.25	*t* = −7.27	< 0.001
CHG‐WHtR, mean ± SD	2.85 ± 0.53	2.82 ± 0.52	2.97 ± 0.57	*t* = −7.23	< 0.001
CHG‐VAI, mean ± SD	12.62 ± 19.37	12.20 ± 19.71	14.09 ± 18.06	*t* = −2.62	0.009
CHG‐CVAI, mean ± SD	503.05 ± 252.26	486.17 ± 249.10	562.40 ± 254.48	*t* = −8.16	< 0.001
cuCHG‐BMI, mean ± SD	372.79 ± 67.68	368.18 ± 66.03	388.98 ± 70.88	*t* = −7.98	< 0.001
cuCHG‐WC, mean ± SD	1334.32 ± 224.33	1318.42 ± 219.70	1390.23 ± 231.50	*t* = −8.65	< 0.001
cuCHG‐WHtR, mean ± SD	8.47 ± 1.44	8.37 ± 1.40	8.83 ± 1.49	*t* = −8.71	< 0.001
cuCHG‐VAI, mean ± SD	35.96 ± 39.69	34.68 ± 39.59	40.49 ± 39.72	*t* = −3.92	< 0.001
cuCHG‐CVAI, mean ± SD	1540.07 ± 708.36	1488.23 ± 700.58	1722.39 ± 705.81	*t* = −8.94	< 0.001
CHG‐BMI cluster, *n* (%)				*χ* ^2^ = 44.40	< 0.001
1	2456 (58.98)	2000 (61.69)	456 (49.46)		
2	1708 (41.02)	1242 (38.31)	466 (50.54)		
CHG‐WC cluster, *n*(%)				*χ* ^2^ = 53.32	< 0.001
1	2189 (52.57)	1802 (55.58)	387 (41.97)		
2	1975 (47.43)	1440 (44.42)	535 (58.03)		
CHG‐WHtR cluster, *n* (%)				*χ* ^2^ = 52.45	< 0.001
1	2230 (53.55)	1833 (56.54)	397 (43.06)		
2	1934 (46.45)	1409 (43.46)	525 (56.94)		
CHG‐VAI cluster, *n* (%)				*χ* ^2^ = 13.13	< 0.001
1	3804 (91.35)	2989 (92.20)	815 (88.39)		
2	360 (8.65)	253 (7.80)	107 (11.61)		
CHG‐CVAI cluster, *n* (%)				*χ* ^2^ = 56.45	< 0.001
1	2286 (54.90)	1880 (57.99)	406 (44.03)		
2	1878 (45.10)	1362 (42.01)	516 (55.97)		
*t*: *t*‐test, *χ* ^2^: chi‐square test					
SD: standard deviation					

*Note:* Continuous variables presented as mean ± standard deviation (mean ± SD); categorical variables expressed as frequencies and percentages (*n* [%]).

Abbreviations: BMI, body mass index; CHG, cholesterol‐high‐density lipoprotein‐glucose; CHG‐ROD, the composite index of CHG and refined obesity derivatives; Cr, creatinine; CVAI, CVD‐associated adiposity index; cu, cumulative; FPG, fasting blood glucose; HDL, high‐density lipoprotein cholesterol; hs‐CRP, high‐sensitivity C‐reactive protein; LDL‐C, low‐density lipoprotein cholesterol; n, number of participants; SD, standard deviation; TC, total cholesterol; VAI, visceral adiposity index; WC, waist circumference; WHtR, waist‐to‐height ratio.

### 3.2. Risk of Incident CVD: Kaplan–Meier Survival Analysis

As illustrated in Figure S1, the risk of incident CVD progressively increased across the ascending quartiles of the CHG‐ROD index. For both baseline and cumulative exposure levels, participants in the highest quartile (Q4) exhibited a significantly elevated risk compared with those in the lowest quartile (Q1) (log − rank *p* < 0.001). Similarly, regarding the trajectory patterns, groups characterized by poor control status across these indices demonstrated an increased risk of incident CVD, a finding that was highly statistically significant (log − rank *p* < 0.001).

### 3.3. Associations of Baseline, Cumulative Exposures, and Trajectories of the CHG‐ROD With Incident CVD

Table 2 details our evaluation of the associations between baseline levels of the composite CHG‐ROD index and incident CVD risk. In the fully adjusted Model 4, the CHG‐ROD indices were significantly and positively associated with incident CVD. Specifically, excluding CHG‐VAI, each 1‐SD increase in the evaluated indices corresponded to a higher risk of incident CVD: CHG‐BMI (19%), CHG‐WC (17%), CHG‐WHtR (14%), and CHG‐CVAI (17%). Further analyses based on baseline quartiles demonstrated that these significant positive associations with CVD risk remained robust.

**Table 2:** Multivariable associations of CHG‐ROD with incident CVD using Cox proportional hazards analysis.

Variables	Model 1		Model 2		Model 3		Model 4	
	HR (95% CI)	*p*	HR (95% CI)	*p*	HR (95% CI)	*p*	HR (95% CI)	*p*
CHG‐BMI (per SD increase)	1.27 (1.20–1.35)	< 0.001	1.30 (1.22–1.38)	< 0.001	1.20 (1.12–1.29)	< 0.001	1.19 (1.11–1.28)	< 0.001
CHG‐BMI quantile								
1	1.00 (reference)		1.00 (reference)		1.00 (reference)		1.00 (reference)	
2	1.18 (0.96–1.45)	0.114	1.22 (0.99–1.50)	0.058	1.16 (0.95–1.43)	0.153	1.15 (0.94–1.42)	0.180
3	1.49 (1.23–1.82)	< 0.001	1.58 (1.29–1.92)	< 0.001	1.41 (1.15–1.73)	< 0.001	1.39 (1.13–1.71)	0.002
4	1.90 (1.57–2.29)	< 0.001	2.01 (1.66–2.43)	< 0.001	1.62 (1.31–1.99)	< 0.001	1.59 (1.28–1.97)	< 0.001
*p* for trend		< 0.001		< 0.001		< 0.001		< 0.001
CHG‐WC (per SD increase)	1.28 (1.20–1.36)	< 0.001	1.27 (1.19–1.36)	< 0.001	1.18 (1.09–1.27)	< 0.001	1.17 (1.08–1.26)	< 0.001
CHG‐WC quantile								
1	1.00 (reference)		1.00 (reference)		1.00 (reference)		1.00 (reference)	
2	1.24 (1.01–1.53)	0.040	1.26 (1.02–1.54)	0.031	1.21 (0.98–1.49)	0.075	1.19 (0.97–1.47)	0.094
3	1.52 (1.24–1.85)	< 0.001	1.52 (1.24–1.85)	< 0.001	1.38 (1.13–1.69)	0.002	1.36 (1.10–1.67)	0.004
4	2.04 (1.69–2.46)	< 0.001	2.03 (1.68–2.45)	< 0.001	1.68 (1.37–2.06)	< 0.001	1.64 (1.34–2.03)	< 0.001
*p* for trend		< 0.001		< 0.001		< 0.001		< 0.001
CHG‐WHtR (per SD increase)	1.28 (1.19–1.36)	< 0.001	1.25 (1.17–1.34)	< 0.001	1.15 (1.07–1.24)	< 0.001	1.14 (1.06–1.23)	< 0.001
CHG‐WHtR quantile								
1	1.00 (reference)		1.00 (reference)		1.00 (reference)		1.00 (reference)	
2	1.24 (1.01–1.52)	0.044	1.24 (1.01–1.52)	0.043	1.20 (0.97–1.47)	0.093	1.18 (0.96–1.46)	0.113
3	1.49 (1.22–1.82)	< 0.001	1.48 (1.21–1.81)	< 0.001	1.34 (1.09–1.65)	0.005	1.31 (1.07–1.62)	0.010
4	2.05 (1.69–2.47)	< 0.001	2.00 (1.64–2.43)	< 0.001	1.64 (1.33–2.02)	< 0.001	1.60 (1.29–1.98)	< 0.001
*p* for trend		< 0.001		< 0.001		< 0.001		< 0.001
CHG‐VAI (per SD increase)	1.06 (1.01–1.11)	0.013	1.06 (1.01–1.11)	0.020	1.02 (0.96–1.08)	0.465	1.04 (0.99–1.11)	0.144
CHG‐VAI quantile								
1	1.00 (reference)		1.00 (reference)		1.00 (reference)		1.00 (reference)	
2	1.30 (1.07–1.59)	0.010	1.29 (1.06–1.58)	0.012	1.24 (1.01–1.52)	0.036	1.23 (1.01–1.51)	0.046
3	1.57 (1.29–1.90)	< 0.001	1.56 (1.28–1.90)	< 0.001	1.41 (1.15–1.72)	< 0.001	1.39 (1.13–1.70)	0.001
4	1.61 (1.33–1.95)	< 0.001	1.60 (1.31–1.95)	<0.001	1.33 (1.08–1.63)	0.007	1.34 (1.09–1.65)	0.006
*p* for trend		< 0.001		< 0.001		0.058		0.042
CHG‐CVAI (per SD increase)	1.30 (1.22–1.39)	< 0.001	1.28 (1.20–1.37)	< 0.001	1.17 (1.09–1.26)	< 0.001	1.17 (1.09–1.26)	< 0.001
CHG‐CVAI quantile								
1	1.00 (reference)		1.00 (reference)		1.00 (reference)		1.00 (reference)	
2	1.33 (1.08–1.64)	0.008	1.29 (1.05–1.60)	0.018	1.25 (1.01–1.55)	0.039	1.24 (1.01–1.54)	0.045
3	1.76 (1.44–2.15)	< 0.001	1.69 (1.38–2.07)	< 0.001	1.55 (1.26–1.90)	< 0.001	1.53 (1.24–1.88)	< 0.001
4	2.20 (1.81–2.67)	< 0.001	2.07 (1.70–2.52)	< 0.001	1.67 (1.35–2.06)	< 0.001	1.66 (1.34–2.05)	< 0.001
*p* for trend		< 0.001		< 0.001		< 0.001		< 0.001
CHG‐BMI cluster								
1	1.00 (reference)		1.00 (reference)		1.00 (reference)		1.00 (reference)	
2	1.50 (1.32–1.71)	< 0.001	1.55 (1.36–1.77)	< 0.001	1.32 (1.15–1.52)	< 0.001	1.30 (1.12–1.50)	< 0.001
CHG‐WC cluster								
1	1.00 (reference)		1.00 (reference)		1.00 (reference)		1.00 (reference)	
2	1.58 (1.39–1.80)	< 0.001	1.57 (1.38–1.79)	< 0.001	1.36 (1.18–1.56)	< 0.001	1.33 (1.15–1.53)	< 0.001
CHG‐WHtR cluster								
1	1.00 (reference)		1.00 (reference)		1.00 (reference)		1.00 (reference)	
2	1.56 (1.37–1.78)	< 0.001	1.53 (1.34–1.75)	< 0.001	1.32 (1.15–1.52)	< 0.001	1.30 (1.12–1.50)	< 0.001
CHG‐VAI cluster								
1	1.00 (reference)		1.00 (reference)		1.00 (reference)		1.00 (reference)	
2	1.40 (1.14–1.71)	0.001	1.39 (1.13–1.70)	0.002	1.21 (0.99–1.50)	0.068	1.26 (1.02–1.56)	0.030
CHG‐CVAI cluster								
1	1.00 (reference)		1.00 (reference)		1.00 (reference)		1.00 (reference)	
2	1.62 (1.42–1.84)	< 0.001	1.55 (1.35–1.76)	< 0.001	1.33 (1.16–1.53)	< 0.001	1.32 (1.14–1.52)	< 0.001
cuCHG‐BMI (per SD increase)	1.27 (1.19–1.35)	< 0.001	1.30 (1.22–1.38)	< 0.001	1.19 (1.11–1.28)	< 0.001	1.18 (1.10–1.27)	< 0.001
cuCHG‐BMI quantile								
1	1.00 (reference)		1.00 (reference)		1.00 (reference)		1.00 (reference)	
2	1.03 (0.84–1.26)	0.795	1.06 (0.87–1.31)	0.550	1.01 (0.82–1.24)	0.934	1.00 (0.81–1.23)	0.997
3	1.36 (1.12–1.65)	0.002	1.43 (1.18–1.74)	< 0.001	1.28 (1.05–1.56)	0.016	1.25 (1.02–1.53)	0.030
4	1.72 (1.43–2.06)	< 0.001	1.82 (1.50–2.19)	< 0.001	1.44 (1.17–1.77)	< 0.001	1.40 (1.14–1.73)	0.001
*p* for trend		< 0.001		< 0.001	)	< 0.001		< 0.001
cuCHG‐WC (per SD increase)	1.31 (1.23–1.39)	< 0.001	1.31 (1.23–1.39)	< 0.001	1.21 (1.13–1.30)	< 0.001	1.20 (1.12–1.29)	< 0.001
cuCHG‐WC quantile								
1	1.00 (reference)		1.00 (reference)		1.00 (reference)		1.00 (reference)	
2	1.25 (1.02–1.54)	0.035	1.27 (1.03–1.57)	0.023	1.23 (1.00–1.51)	0.052	1.22 (0.99–1.51)	0.061
3	1.62 (1.33–1.97)	< 0.001	1.63 (1.34–1.99)	< 0.001	1.48 (1.21–1.81)	< 0.001	1.46 (1.19–1.79)	< 0.001
4	1.99 (1.65–2.41)	< 0.001	1.99 (1.65–2.41)	< 0.001	1.63 (1.33–2.00)	< 0.001	1.60 (1.30–1.96)	< 0.001
*p* for trend		< 0.001		< 0.001		< 0.001		< 0.001
cuCHG‐WHtR (per SD increase)	1.31 (1.23–1.39)	< 0.001	1.29 (1.21–1.38)	<0.001	1.19 (1.11–1.28)	< 0.001	1.18 (1.09–1.27)	< 0.001
cuCHG‐WHtR quantile								
1	1.00 (reference)		1.00 (reference)		1.00 (reference)		1.00 (reference)	
2	1.26 (1.02–1.54)	0.031	1.27 (1.03–1.56)	0.024	1.22 (0.99–1.50)	0.067	1.21 (0.98–1.49)	0.076
3	1.57 (1.29–1.91)	< 0.001	1.58 (1.29–1.93)	< 0.001	1.42 (1.16–1.74)	< 0.001	1.39 (1.13–1.71)	0.002
4	2.01 (1.67–2.44)	< 0.001	1.98 (1.63–2.41)	< 0.001	1.60 (1.29–1.97)	< 0.001	1.56 (1.26–1.93)	< 0.001
*p* for trend		< 0.001		< 0.001		< 0.001		< 0.001
cuCHG‐VAI (per SD increase)	1.09 (1.04–1.15)	< 0.001	1.09 (1.04–1.15)	< 0.001	1.04 (0.99–1.11)	0.135	1.07 (1.01–1.13)	0.036
cuCHG‐VAI quantile								
1	1.00 (reference)		1.00 (reference)		1.00 (reference)		1.00 (reference)	
2	1.27 (1.04–1.56)	0.021	1.30 (1.05–1.59)	0.014	1.23 (1.01–1.52)	0.046	1.22 (0.99–1.50)	0.063
3	1.62 (1.33–1.96)	< 0.001	1.63 (1.34–2.00)	< 0.001	1.45 (1.18–1.77)	< 0.001	1.43 (1.16–1.75)	< 0.001
4	1.72 (1.42–2.08)	< 0.001	1.76 (1.44–2.15)	< 0.001	1.45 (1.18–1.79)	< 0.001	1.45 (1.17–1.79)	< 0.001
*p* for trend		< 0.001		< 0.001		0.003		0.003
cuCHG‐CVAI (per SD increase)	1.33 (1.25–1.42)	< 0.001	1.30 (1.22–1.39)	< 0.001	1.19 (1.11–1.28)	< 0.001	1.19 (1.11–1.28)	< 0.001
cuCHG‐CVAI quantile								
1	1.00 (reference)		1.00 (reference)		1.00 (reference)		1.00 (reference)	
2	1.45 (1.17–1.79)	< 0.001	1.42 (1.15–1.76)	0.001	1.38 (1.11–1.71)	0.003	1.37 (1.11–1.70)	0.004
3	1.82 (1.48–2.23)	< 0.001	1.75 (1.42–2.15)	< 0.001	1.60 (1.30–1.97)	< 0.001	1.58 (1.28–1.95)	< 0.001
4	2.29 (1.89–2.79)	< 0.001	2.17 (1.77–2.64)	< 0.001	1.75 (1.41–2.16)	< 0.001	1.73 (1.40–2.14)	< 0.001
*p* for trend		< 0.001		< 0.001		< 0.001		< 0.001

*Note:* Model 1 was unadjusted; Model 2 was adjusted for age and gender; Model 3 was further adjusted for drinking status, smoking status, hypertension, diabetes, marital status, education, and dyslipidemia based on Model 2. Model 4 was additionally adjusted for Cr and hs‐CRP based on Model 3.

Abbreviations: CI, confidence interval; CVD, cardiovascular disease; HR, hazard ratios; other abbreviations as in Table 1..

Table 2 also presents the associations between the cumulative exposures to these indices and incident CVD. In the fully adjusted Model 4, the cumulative exposures of the composite indices exhibited significant positive associations with incident CVD. Specifically, each 1‐SD increment in the composite indices (cuCHG‐BMI, cuCHG‐WC, cuCHG‐WHtR, cuCHG‐VAI, and cuCHG‐CVAI) was associated with an 18%, 20%, 18%, 7%, and 19% higher risk of incident CVD, respectively. Moreover, further categorical analyses utilizing the quartiles of these cumulative exposures yielded consistent positive associations.

Furthermore, Table 2 delineates the associations of the distinct trajectory patterns of these indices with incident CVD. In the fully adjusted Model 4, compared with the well‐controlled reference group, participants with poorly controlled trajectories for the composite indices (CHG‐BMI, CHG‐WC, CHG‐WHtR, CHG‐VAI, and CHG‐CVAI) had a significantly increased risk of incident CVD.

### 3.4. Dose–Response Relationships

Figure 5A illustrates the dose–response relationships of the baseline levels of the composite indices with incident CVD. After full adjustment for potential confounders, linear relationships were observed for baseline levels of the composite indices (CHG‐BMI and CHG‐CVAI) (*p* for nonlinearity > 0.05). Conversely, the baseline composite indices (CHG‐WC, CHG‐WHtR, and CHG‐VAI) exhibited non‐linear trends (*p* for non − linearity < 0.05).

**Figure 5:**
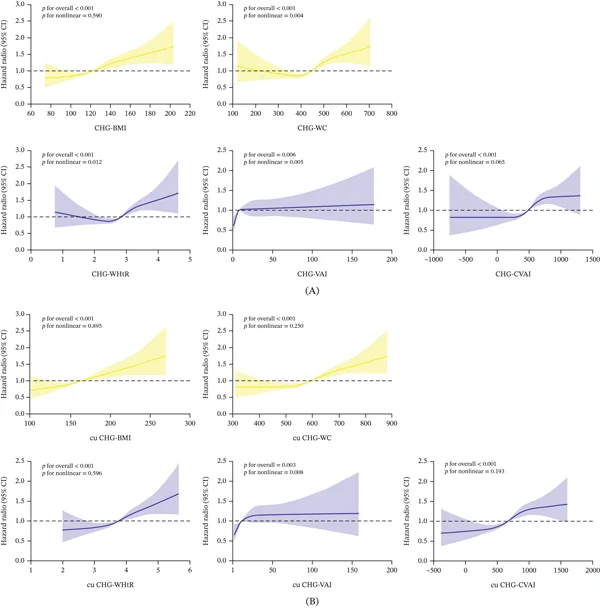
Dose–response of baseline/cumulative CHG‐ROD to CVD risk using restricted cubic spline. (A) Dose–response relationship of the baseline levels of CHG‐ROD with incident CVD; (B) dose–response relationships of cumulative exposures of CHG‐ROD with incident CVD. CHG, cholesterol‐high‐density lipoprotein‐glucose; CHG‐ROD, the composite index of CHG and refined obesity derivatives; WC, waist circumference; WHtR, waist‐to‐height ratio; BMI, body mass index; VAI, visceral adiposity index; and CVAI, CVD‐associated adiposity index.

Similarly, Figure 5B depicts the dose–response relationships of cumulative exposures to these indices with incident CVD. Following full adjustment, cumulative exposures to most composite indices (cuCHG‐BMI, cuCHG‐WC, cuCHG‐WHtR, and cuCHG‐CVAI) demonstrated linear associations (*p* for non − linearity > 0.05). In contrast, only the cumulative exposure to the CHG‐VAI index displayed a nonlinear relationship (*p* for nonlinearity < 0.05).

### 3.5. Predictive Performance Analysis

As shown in Table S3, ROC curve analysis revealed that the predictive capabilities for incident CVD of the baseline composite indices (CHG‐BMI, CHG‐WC, CHG‐WHtR, and CHG‐CVAI) were significantly superior to that of the CHG index alone (all DeLong *p* < 0.05). The area under the curve (AUC) of the composite CHG‐VAI index was lower than that of the CHG index alone, though also without statistical significance (DeLong *p* > 0.05). Notably, the composite CHG‐CVAI and CHG‐WC indices demonstrated the highest predictive accuracy.

As detailed in Table S4, ROC curve analysis of the cumulative exposures indicated that the predictive capabilities of the composite indices (cuCHG‐WC, cuCHG‐WHtR, and cuCHG‐CVAI) were significantly superior to that of the cuCHG index alone (all DeLong *p* < 0.05). The AUC of the composite cuCHG‐BMI was higher than that of the cuCHG index alone, albeit not statistically significant (DeLong *p* > 0.05). The AUC of the composite cuCHG‐VAI indices was lower than that of the cuCHG index alone (DeLong *p* > 0.05). Among all cumulative indices assessed, cuCHG‐CVAI and cuCHG‐WC exhibited the highest predictive performance.

As presented in Table S5, the incorporation of the composite indices (both baseline and cumulative exposure levels) into the FRS model yielded a significant enhancement in predictive performance for incident CVD.

Although the absolute gains in AUC were modest, all augmented models had significantly higher AUC than the FRS model alone, with supporting evidence from NRI and IDI analyses.

### 3.6. WQS Regression Analysis

A WQS regression model was employed to quantify the differential contributions of cumulative exposure components within the composite indices to the risk of incident CVD. The analysis revealed that the empirical weights assigned to cumulative obesity‐related indices were substantially higher than those of other metabolic parameters (Figure 6). This finding underscores that long‐term adiposity serves as a core driving factor in the pathogenesis of CVD.

**Figure 6:**
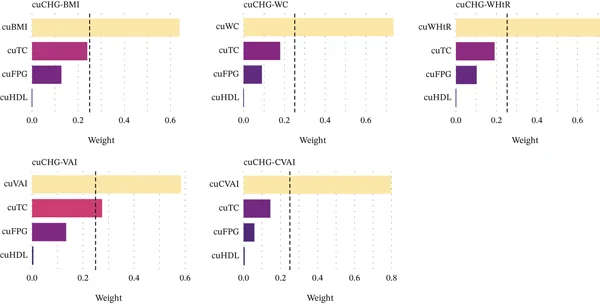
Component contribution rates in cumulative CHG‐ROD via WQS regression. Cumulative CHG‐ROD composite indices (cuCHG‐BMI, cuCHG‐WC, cuCHG‐WHtR, cuCHG‐VAI, and cuCHG‐CVAI). CHG, cholesterol‐high‐density lipoprotein‐glucose; CHG‐ROD, the composite index of CHG and refined obesity derivatives; CVD, cardiovascular disease; WQS, weighted quantile sum; cu, cumulative; FPG, fasting blood glucose; TC, total cholesterol; HDL, high‐density lipoprotein cholesterol; LDL‐C, low‐density lipoprotein cholesterol; WC, waist circumference; WHtR, waist‐to‐height ratio; BMI, body mass index; VAI, visceral adiposity index; and CVAI, CVD‐associated adiposity index.

### 3.7. Sensitivity Analyses

First, subgroup analyses were conducted to explore the associations of the baseline levels, cumulative exposures, and longitudinal trajectories of the composite indices with incident CVD across diverse subpopulations. The results demonstrated that across the majority of subgroups, the baseline levels, cumulative exposures, and longitudinal trajectories of these indices remained significantly associated with an increased risk of incident CVD. Significant interactions with age regarding incident CVD risk were observed only for the baseline cuCHG‐WHtR and the trajectory patterns of CHG‐WHtR. Additionally, a significant interaction was detected between the baseline CHG‐VAI and smoking status. Furthermore, the cuCHG‐CVAI and the trajectory patterns of the CHG‐BMI exhibited significant interactions with hypertension status. (Figure S2A, Figure S2B, Table S6). Second, given the possibility that recently developed CVD events might reversely affect the composite indices, we excluded participants who were diagnosed with CVD during the third and fourth survey waves. Upon repeating the association analyses, the findings remained highly consistent with the primary results, further confirming their robustness (Table S7).

## 4. Discussion

The present study systematically explored the associations of the baseline levels, cumulative exposures, and longitudinal trajectory patterns of the composite CHG‐ROD indices with the risk of incident CVD. Our results demonstrated that these indices—across baseline, cumulative, and trajectory dimensions—were significantly associated with the incidence of CVD, and these associations remained robust even after multivariable adjustment. Regarding predictive performance, the composite CHG‐ROD indices—particularly the baseline and cumulative exposure levels of CHG‐WC and CHG‐CVAI—were significantly superior to the CHG index alone. Furthermore, the incorporation of these indices into the FRS model yielded a significant enhancement in its predictive capability. We observed significant gains in AUC compared with the CHG index or FRS model alone, although the absolute increments were modest. NRI and IDI provided complementary evidence of improved risk reclassification despite the limited absolute change in AUC. This indicates that these novel composite indices provide additional prognostic information independent of traditional FRS risk factors. Consequently, the composite CHG‐ROD indices hold great promise for refining CVD risk stratification. Extensive sensitivity analyses further corroborated the robustness of these findings.

The CHG index, originally proposed by Mansoori et al. (15), is an integrated index based on total TC, HDL, and FBG. It was originally developed to serve as a readily available surrogate marker for the homeostasis model assessment of IR, which is less accessible in routine clinical practice. Notably, the CHG index has been shown to exhibit superior specificity and predictive performance for Type 2 diabetes compared with the TyG index (15, 23). Furthermore, previous studies have validated its important clinical implications for CVD risk stratification among middle‐aged and older Chinese adults (16). Building on these findings, Degang Mo et al. (17). further explored the predictive value of the CHG index for CVD risk in comparison with the TyG index. They demonstrated that a higher CHG index was significantly and linearly associated with an increased risk of CVD, with predictive efficacy comparable to that of the TyG index and consistent across diverse subgroups. However, the CHG index alone is confined to parameters of glucolipid metabolism, lacking the incorporation of anthropometric measures—such as BMI and WC—that reflect adiposity accumulation and fat distribution. Given that obesity is intricately linked to CVD pathogenesis through the exacerbation of IR and the induction of chronic inflammatory responses, the CHG index alone falls short of fully capturing the synergistic pathogenic risk driven by the coexistence of glucolipid metabolic disorders and adiposity. Indeed, a similar rationale for synergistic enhancement has been validated in studies of other IR surrogates (24, 25): when the TyG index is combined with obesity indices, the predictive performance is significantly superior to that of the IR marker alone. This precedent strongly underscores the necessity of integrating the CHG index with obesity‐related derivatives to overcome its inherent limitations.

In clinical practice and research, BMI is frequently utilized as the core index for assessing obesity because of its measurement convenience and data accessibility. However, it merely reflects the relative relationship between weight and height; its fundamental limitation lies in the inability to differentiate fat distribution (e.g., the more pathogenic visceral fat vs. the relatively benign subcutaneous fat). Previous studies (26) have demonstrated a U‐shaped nonlinear relationship between BMI and mortality among patients with CVD. Conversely, when assessed using WC, mortality exhibits a linear positive correlation (27, 28). This suggests that the so‐called “obesity paradox” likely stems from the definitional flaws of BMI regarding adiposity, rather than obesity inherently conferring a protective effect. Given that visceral fat accumulation is the key pathological foundation driving elevated CVD risk, traditional BMI lacks the specificity to distinguish fat distribution, rendering it inadequate to accurately capture this core risk dimension.

Therefore, there is an urgent need to introduce refined obesity derivatives capable of specifically characterizing highly pathogenic visceral adiposity, thereby optimizing the accuracy and clinical utility of CVD risk assessment. In recent years, a variety of refined obesity derivatives have been successively proposed. These derivatives (19, 20, 29) not only possess high feasibility in routine clinical practice but also more precisely characterize the distributional patterns of human fat accumulation (particularly visceral fat). Multiple studies have highlighted that composite indices of the TyG index and various obesity‐related indices are significantly associated with CVD events, and their predictive performances are generally superior to that of the TyG index alone. A 9‐year national cohort study (30) focusing on populations with Stage 0–3 cardiovascular‐kidney‐metabolic syndrome revealed that the composite indices of TyG‐BMI, TyG‐WC, and TyG‐WHtR were linearly and positively associated with the risk of incident CVD, with TyG‐WC and TyG‐WHtR exhibiting the optimal predictive performance. Similarly, research involving middle‐aged and older populations (31) confirmed that the predictive efficacy of the composite index of TyG‐CVAI for incident CVD was significantly superior to that of the TyG or CVAI alone. A 12‐year cohort study (32) of postmenopausal Chinese women demonstrated that the composite index of TyG‐WC was the strongest predictor for CVD, whereas the composite index of TyG‐WHtR metric showed the optimal diagnostic value; both significantly enhanced the risk stratification capacity of the models. Consistent with previous findings, our study highlights the superiority of the CHG‐based composite indices—specifically CHG‐WC and CHG‐CVAI—as exceptional tools for CVD risk assessment that perfectly balance clinical simplicity with robust predictive performance.

Although IR surrogate index and composite of IR‐obesity derivative indices have demonstrated robust predictive value for CVD, further integrating their longitudinal trajectory patterns and cumulative exposures effectively overcomes the limitations of cross‐sectional measurements. This strategy enables a more comprehensive capture of the persistent, synergistic risk driven by the long‐term coexistence of IR and adiposity, thereby significantly enhancing the performance of CVD prediction models. Consistent with our findings, Wang et al. (21), systematically evaluated the impact of the longitudinal trajectory patterns, specifically incorporating cumulative exposure levels and K‐Means‐based trajectory partitioning of the composite indices of TyG and obesity derivatives on CVD events. Their study confirmed that this longitudinal dynamic evaluation strategy offered significant predictive advantages over static baseline assessments, demonstrating superior predictive performance.

Currently, the exact pathophysiological mechanisms underlying the predictive value of the composite indices of CHG‐ROD for CVD risk remain to be fully elucidated. Most scholars posit that IR (33, 34) acts as the central hub, with glucolipid metabolism (35, 36), inflammatory responses (37), and oxidative stress (38, 39) serving as potential underlying mechanisms. In the state of abdominal adiposity, adipose tissue abnormally secretes excessive amounts of free fatty acids (FFAs), reactive oxygen species (ROS), and pro‐inflammatory cytokines. The surplus circulating FFAs undergo ectopic deposition in nonadipose tissues and are metabolized into lipotoxic mediators such as ceramides. The accumulation of these molecules in mitochondria and the endoplasmic reticulum triggers organelle stress and dysfunction, leading to target cell injury and systemic metabolic dyshomeostasis (40). This metabolic perturbation significantly blunts the insulin sensitivity of target organs and disrupts glucose homeostasis, thereby triggering IR. Subsequently, IR—characterized by compensatory hyperinsulinemia—further deranges the body′s glucolipid metabolism and neuroendocrine regulatory networks, exacerbating the excessive accumulation and ectopic distribution of fat. This pathological process stimulates the continuous generation of massive ROS and pro‐inflammatory cytokines (41), inducing a state of systemic low‐grade inflammation. Within this microenvironment, immune cells undergo chemotactic infiltration into adipose tissue and damaged target organs. Their aberrant activation fails to restore tissue homeostasis; instead, it further amplifies local and systemic inflammatory cascades (41), thereby forging a vicious cycle of “obesity and IR” (42). Ultimately, the superimposed pathogenic effects of IR and adiposity collectively drive chronic low‐grade inflammation (43), vascular endothelial injury (44), and mitochondrial dysfunction, constituting the core pathological foundation for the onset and progression of CVD.

The subgroup analyses revealed that, across baseline levels, cumulative exposures, and longitudinal trajectory patterns, the composite indices of CHG‐WC and CHG‐CVAI exhibited no significant interactions across the vast majority of subpopulations. However, a significant interaction was uniquely identified between the cuCHG‐CVAI and incident CVD risk concerning hypertension status. Specifically, within the hypertensive subgroup, the strength of the association between the cuCHG‐CVAI and incident CVD was markedly attenuated. We postulate that this interaction effect is inextricably linked to the inherent pathophysiological mechanisms of hypertension: first, as a potent independent risk factor for CVD, hypertension and its chronic hemodynamic abnormalities precipitate a well‐defined cascade of target organ damage, including the impairment of the vascular endothelial barrier, smooth muscle proliferation, and vascular remodeling (45). Within the hypertensive subgroup, these profound pathological alterations may constitute the dominant driving force for incident CVD, thereby masking the incremental predictive value of the comprehensive metabolic risk encapsulated by the cuCHG‐CVAI. Second, hypertensive patients typically undergo standardized antihypertensive regimens and comprehensive cardiovascular management. Beyond blood pressure control, such clinical interventions may concomitantly ameliorate other metabolic risk factors encompassed by the cuCHG‐CVAI. This synergistic improvement could dilute the risk associated with long‐term cumulative exposure (46), resulting in a blunted association with incident CVD. Nevertheless, constrained by the sample size of the hypertensive subgroup in the present study, these interpretations of the interaction effect should be approached with caution.

Of note, CHG‐VAI exhibited inferior predictive performance relative to other CHG‐ROD composites. Several plausible reasons may account for this finding. First, the VAI equation was initially established in Western populations with ethnically tuned coefficients, which might not perfectly characterize visceral adiposity dysfunction among Chinese middle‐aged and older adults, unlike CVAI specifically developed for Chinese populations. Second, in our cohort, nearly 91.35% of participants maintained well‐controlled CHG‐VAI status, yielding highly imbalanced trajectory group sizes that weaken risk discrimination. Consistently, baseline CHG‐VAI showed no significant independent association with CVD risk in fully‐adjusted Cox models. Third, VAI already embeds lipid‐related parameters overlapping with the CHG index; combining them introduces redundant metabolic information and provides limited additional predictive signal. Moreover, VAI serves as an indirect surrogate of visceral fat rather than a direct phenotypic metric, so it cannot adequately capture the core pathogenic burden of abdominal adiposity. Together, these factors may explain the reduced predictive efficiency of CHG‐VAI, further supporting that CHG‐WC and CHG‐CVAI represent more appropriate composite markers for CVD risk prediction in this Chinese middle‐aged and elderly population.

Drawing upon data from a national prospective cohort, our study represents the first systematic delineation of the dynamic evolution of the composite indices of CHG‐ROD, uncovering their profound associations with incident cardiovascular events. Our findings highlight the superiority of the longitudinal composite indices constructed in this study. Compared with single indicators, these composite indices incorporate multidimensional information, providing a more comprehensive characterization of glucolipid metabolic dyshomeostasis and abdominal adiposity. Furthermore, moving beyond static, cross‐sectional assessments, the present study captured longitudinal trajectories. This approach enables a more accurate and dynamic evaluation of the cumulative exposure burden driving adverse outcomes. Targeted interventions against abdominal adiposity—particularly the effective reduction of WC—can yield tangible cardiovascular benefits by rectifying underlying metabolic dyshomeostasis and suppressing chronic inflammatory cascades. In conclusion, tracking the longitudinal trajectories of the CHG‐ROD composite indices holds immense potential for clinical translation, serving as a dynamic tool to guide these preventive strategies.

Several limitations of the present study warrant consideration. First, although we maximized the utility of available longitudinal data to perform cumulative exposure and trajectory analyses, data acquisition for the CHG index and CHG‐ROD indices (e.g., WC) relied on discrete measurements at specific follow‐up time points. Consequently, these indices may not fully capture the continuous, dynamic true variations occurring within individuals over the long‐term exposure period. Second, WC measurements are susceptible to procedural variations and subject posture, inevitably introducing a degree of measurement bias. Thirdly, the ascertainment of incident CVD events in this study was primarily based on self‐reported medical histories, health insurance records, and death registries, lacking uniform adjudication against strict clinical gold standards. This reliance may introduce recall bias and potential outcome misclassification bias, thereby compromising the precision of the estimated exposure‐outcome associations (47). Fourthly, our two‐cluster grouping has notable limitations. Binary classification into well‐controlled and poorly controlled trajectories may oversimplify the continuous spectrum of CHG‐ROD longitudinal variation and ignore intermediate trajectory phenotypes. Since trajectory modeling was restricted to two time points, we could not identify more complex dynamic patterns of CHG‐ROD change. Although the *k* = 2 solution was statistically optimal and clinically interpretable, this dichotomous approach limits our capacity to examine graded CVD risk across a wider range of longitudinal CHG‐ROD patterns. Future studies with repeated biomarker measurements over more survey waves are needed to characterize finer trajectory subtypes. Additionally, the study cohort was restricted to middle‐aged and older Chinese adults aged 45 years and older, which limits the generalizability of our findings to other ethnic populations or age demographic groups. Finally, the relatively limited sample size for certain extreme metabolic phenotypes attenuated the statistical power of our subgroup analyses to some extent.

Our study boasts several notable strengths. First, to address the limitations of the traditional FRS in fully capturing IR and central adiposity, we explored and validated the enhanced predictive capacity of using the CHG‐ROD composite indices alongside the FRS. Incorporating the composite indices significantly augmented the predictive and reclassification capabilities of the traditional FRS model, yielding essential insights for more precise CVD risk stratification. Second, the introduction of the WQS regression model allowed for the precise quantification of the differential contributions of various cumulative exposure components within the CHG‐ROD composite indices to incident CVD. This further corroborated the pivotal role of long‐term lipid metabolic perturbations and central adiposity in driving the onset and progression of CVD. Finally, regarding the longitudinal design, the period from 2012 to 2015 was strictly designated as the evaluation window for exposure trajectories, and the follow‐up time point furthest from this window was deliberately selected as the observation endpoint for incident CVD. This rigorous timeline design effectively circumvented the reverse causality bias that often arises when exposure and outcome occur in close temporal proximity. By ensuring that the establishment of dynamic exposure trajectories completely preceded the occurrence of clinical outcomes, this approach methodologically maximized the chronological rigor and causal inference capacity of our findings.

## 5. Conclusion

In conclusion, this cohort study demonstrates that the longitudinal trajectories of a composite lipid, glucose, and refined obesity index are reliable predictors of incident CVD. Specifically, composite indices incorporating WC (CHG‐WC) and cardiovascular‐associated adiposity (CHG‐CVAI) exhibited superior predictive performance. These findings suggest that elevated cardiovascular risk is primarily driven by the synergistic pathogenic effects of IR and abdominal adiposity.

## Author Contributions

L.X., T.J., W.W., and J.D.: conceptualization, methodology. L.X.: visualization, resources, and investigation. W.W. and J.D.: supervision and project administration. L.X. and T.J.: writing—original draft preparation. Y.W., W.W., D.L., and Y.S.: software, formal analysis, and validation. L.X., T.J., and Y.W.: data curation. L.X., T.J., Y.W., D.L., Y.S., W.W., and J.D.: writing—reviewing and editing. L.X. and T.J. contributed equally.

## Funding

No funding was received for this manuscript.

## Disclosure

All authors read and approved the final manuscript.

## Ethics Statement

This study used public anonymous data from the China Health and Retirement Longitudinal Study (CHARLS). All participants provided written informed consent, and the CHARLS protocol was approved by the Institutional Review Board of Peking University (Approval Number: IRB00001052‐11015). This study strictly adhered to the ethical principles outlined in the Declaration of Helsinki, and the reporting of results complies with the Strengthening the Reporting of Observational Studies in Epidemiology (STROBE) guidelines.

## Consent

The authors have nothing to report.

## Conflicts of Interest

The authors declare no conflicts of interest.

## Supporting information


**Supporting Information** Additional supporting information can be found online in the Supporting Information section. File S1: Calculation methods of cumulative CHG‐ROD. Table S1: K‐means clustering silhouette coefficient based on CHG‐ROD changes. Table S2: Multicollinearity assessment (VIF) of Model 4 variables. Figure S1A: Kaplan–Meier analysis: CHG‐ROD quartiles and incident CVD. Figure S1B: Kaplan–Meier analysis: cumulative CHG‐ROD and incident CVD. Figure S1C: KM analysis: trajectory pattern of CHG‐ROD and incident CVD. Table S3: ROC curve analysis of baseline CHG and CHG‐ROD in predicting incident CVD. Table S4: ROC curve analysis of cumulative CHG and CHG‐ROD in predicting incident CVD. Table S5: Predictive performance of the FRS model incorporating CHG‐ROD. Figure S2A: Subgroup analyses of baseline CHG‐ROD in relation to CVD risk. Figure S2B: Subgroup analyses of cumulative CHG‐ROD in relation to CVD risk. Table S6: Subgroup analyses of trajectory CHG‐ROD in relation to CVD risk. Table S7: Association of CHG‐ROD with CVD risk after excluding participants with recent CVD.

## Data Availability

The data that support the findings of this study are available in CHARLS at http://charls.pku.edu.cn/en. These data were derived from the following resources available in the public domain: CHARLS, http://charls.pku.edu.cn/en.
