# Supplementary material for: Trajectories of a Composite Lipid, Glucose, and Refined Obesity Index Predict Cardiovascular Disease
Source: Cardiovasc Ther. 2026 Nov 4;2026:1092234. doi: 10.1155/cdr/1092234 (PMC13732808; doi:10.1155/cdr/1092234)
Supplement: Supplementary file 1 [file CDR-2026-1092234-s001.docx]

**Supplementary File1**.Calculation methods of cumulative CHG-ROD

CHG - ROD = CHG $\times$ ROD.

$$Cumulative (CHG-ROD index)=\frac{(CHG\times ROD 2012+ CHG\times ROD 2015)}{2}\times duration（2015-2012）$$

$$Cumulative (CHG-BMI index)=\frac{(CHG-BMI index 2012+ CHG-BMI index 2015)}{2}\times\mathrm{duration}（2015-2012）$$

$$Cumulative (CHG-WC index)=\frac{(CHG-WC index 2012+ CHG-WC index 2015)}{2}\times\mathrm{duration}（2015-2012）$$

$$Cumulative (CHG-WHtR index)=\frac{(CHG-WHtR index 2012+ CHG-WHtR index 2015)}{2}\times\mathrm{duration}（2015-2012）$$

$$Cumulative (CHG-VAI index)=\frac{(CHG-VAI index 2012+ CHG-VAI index 2015)}{2}\times\mathrm{duration}（2015-2012）$$

$$Cumulative (CHG-CVAI index)=\frac{(CHG-CVAI index 2012+ CHG-CVAI index 2015)}{2}\times\mathrm{duration}（2015-2012）$$

CHG: cholesterol-high-density lipoprotein-glucose; CHG-ROD: the composite index of CHG and refined obesity derivatives; WC: waist circumference; WHtR: waist-to-height ratio; BMI: body mass index; VAI: visceral adiposity index; CVAI: CVD-associated adiposity index.

**Supplementary Table1.** K-means Clustering Silhouette Coefficient Based on CHG - ROD Changes

| Variables | Silhouette Coefficient |
| --- | --- |
| CHG-BMI | 0.61 |
| CHG-WC | 0.55 |
| CHG-WHtR | 0.56 |
| CHG-VAI | 0.86 |
| CHG-CVAI | 0.57 |

CHG: cholesterol-high-density lipoprotein-glucose; WC: waist circumference; WHtR: waist-to-height ratio; BMI: body mass index; VAI: visceral adiposity index; CVAI: CVD-associated adiposity index.

**Supplementary Table2.** Multicollinearity Assessment (VIF) of Model 4 Variables

| Variables | VIF |
| --- | --- |
| Age | 1.092 |
| Gender | 1.480 |
| Drinking status | 1.161 |
| Smoking status | 1.264 |
| Marital status | 1.044 |
| Education | 1.018 |
| Hypertension | 1.048 |
| Diabetes | 1.155 |
| Dyslipidemia | 1.028 |
| Cr | 1.172 |
| hs-CRP | 1.172 |
| CHG-BMI | 1.368 |
| CHG-WC | 1.274 |
| CHG-WHtR | 1.298 |
| CHG-VAI | 1.161 |
| CHG-CVAI | 1.258 |
| cuCHG-BMI | 1.360 |
| cuCHG-WC | 1.323 |
| cuCHG-WHtR | 1.363 |
| cuCHG-VAI | 1.264 |
| cuCHG-CVAI | 1.279 |
| CHG-BMI Cluster | 1.211 |
| CHG-WC Cluster | 1.240 |
| CHG-WHtR Cluster | 1.244 |
| CHG-VAI Cluster | 1.155 |
| CHG-CVAI Cluster | 1.205 |

CHG: cholesterol-high-density lipoprotein-glucose; CHG-ROD: the composite index of CHG and refined obesity derivatives; VIF: variance inflation factors; Cr: creatinine; hs-CRP: high-sensitivity c-reactive protein; cu: cumulative; WC: waist circumference; WHtR: waist-to-height ratio; BMI: body mass index; VAI: visceral adiposity index; CVAI: CVD-associated adiposity index.

**Supplementary Figure1A.** **Kaplan–Meier analysis: CHG-ROD quartiles and incident CVD**

CHG: cholesterol-high-density lipoprotein-glucose; CHG-ROD: the composite index of CHG and refined obesity derivatives; CVD: cardiovascular disease; WC: waist circumference; WHtR: waist-to-height ratio; BMI: body mass index; VAI: visceral adiposity index; CVAI: CVD-associated adiposity index.

**Supplementary Figure1B. Kaplan–Meier analysis: cumulative CHG-ROD and incident CVD**

CHG: cholesterol-high-density lipoprotein-glucose; CHG-ROD: the composite index of CHG and refined obesity derivatives; CVD: cardiovascular disease; WC: waist circumference; WHtR: waist-to-height ratio; BMI: body mass index; VAI: visceral adiposity index; CVAI: CVD-associated adiposity index.

**Supplementary Figure1C.KM analysis: trajectory pattern of CHG-ROD and incident CVD**

CHG: cholesterol-high-density lipoprotein-glucose; CHG-ROD: the composite index of CHG and refined obesity derivatives; CVD: cardiovascular disease; WC: waist circumference; WHtR: waist-to-height ratio; BMI: body mass index; VAI: visceral adiposity index; CVAI: CVD-associated adiposity index.

**Supplementary Table3** ROC curve analysis of baseline CHG and CHG-ROD in predicting incident CVD

| Variables | AUC | 95% CI | Specificity | Sensitivity | Cut-off |
| --- | --- | --- | --- | --- | --- |
| CHG | 0.571 | 0.550 ~ 0.592 | 0.577 | 0.538 | 5.294 |
| CHG-BMI* | 0.589 | 0.568 ~ 0.610 | 0.484 | 0.661 | 131.260 |
| CHG-WC* | 0.594 | 0.573 ~ 0.615 | 0.656 | 0.491 | 438.861 |
| CHG-WHtR* | 0.592 | 0.571 ~ 0.612 | 0.501 | 0.640 | 2.971 |
| CHG-VAI | 0.564 | 0.543 ~ 0.584 | 0.703 | 0.401 | 5.917 |
| CHG-CVAI* | 0.597 | 0.577 ~ 0.618 | 0.672 | 0.489 | 457.670 |

Asterisks indicate an increase in the AUC. CHG: cholesterol-high-density lipoprotein-glucose; CHG-ROD: the composite index of CHG and refined obesity derivatives; CVD: cardiovascular disease; AUC: area under the curve; CI: confidence interval; WC: waist circumference; WHtR: waist-to-height ratio; BMI: body mass index; VAI: visceral adiposity index, and CVAI: CVD-associated adiposity index.

**Supplementary Table4** ROC curve analysis of cumulative CHG and CHG-ROD in predicting incident CVD

| Variables | AUC | 95% CI | Specificity | Sensitivity | Cut-off |
| --- | --- | --- | --- | --- | --- |
| cuCHG | 0.572 | 0.551 ~ 0.593 | 0.600 | 0.521 | 6.947 |
| cuCHG-WC* | 0.594 | 0.574 ~ 0.615 | 0.716 | 0.433 | 565.982 |
| cuCHG-BMI | 0.586 | 0.565 ~ 0.607 | 0.566 | 0.564 | 165.595 |
| cuCHG-WHtR* | 0.592 | 0.571 ~ 0.613 | 0.653 | 0.494 | 3.678 |
| cuCHG-VAI | 0.571 | 0.551 ~ 0.592 | 0.586 | 0.534 | 11.263 |
| cuCHG-CVAI* | 0.601 | 0.580 ~ 0.622 | 0.777 | 0.380 | 554.344 |

CHG: cholesterol-high-density lipoprotein-glucose; CHG-ROD: the composite index of CHG and refined obesity derivatives; CVD: cardiovascular disease; cu, cumulative; AUC: area under the curve; CI: confidence interval; WC: waist circumference; WHtR: waist-to-height ratio; BMI: body mass index; VAI: visceral adiposity index, and CVAI: CVD-associated adiposity index.

**Supplementary Table5** Predictive performance of the FRS model incorporating CHG-ROD

| Variables | C Statistics | AUC | | NRI | | IDI | |
| --- | --- | --- | --- | --- | --- | --- | --- |
|  | Estimate(95%CI) | Estimate(95%CI) | DeLong- P value | Estimate(95%CI) | P value | Estimate(95%CI) | P value |
| FRS | 0.575 (0.556-0.594) | 0.605 (0.563-0.646) | Reference | Reference | Reference | Reference | Reference |
| FRS+CHG-BMI | 0.602 (0.583-0.621) | 0.621 (0.580-0.662) | ＜0.001 | 0.230 (0.146-0.314) | ＜0.001 | 0.009 (0.006-0.012) | ＜0.001 |
| FRS+CHG-WC | 0.597 (0.578-0.616) | 0.612 (0.571-0.653) | ＜0.001 | 0.173 (0.089-0.257) | ＜0.001 | 0.006 (0.004-0.009) | ＜0.001 |
| FRS+CHG-WHtR | 0.600 (0.581-0.619) | 0.617 (0.576-0.657) | ＜0.001 | 0.203 (0.119-0.287) | ＜0.001 | 0.007 (0.004-0.010) | ＜0.001 |
| FRS+CHG-VAI | 0.578 (0.559-0.597) | 0.608 (0.568-0.649) | 0.026 | 0.097 (0.019-0.174) | 0.014 | 0.001 (0.000-0.001) | 0.004 |
| FRS+CHG-CVAI | 0.600 (0.581-0.619) | 0.626 (0.586-0.666) | ＜0.001 | 0.212 (0.128-0.296) | ＜0.001 | 0.007 (0.004-0.009) | ＜0.001 |
| FRS+cuCHG- BMI | 0.602 (0.583-0.621) | 0.624 (0.583-0.665) | ＜0.001 | 0.199 (0.115-0.283) | ＜0.001 | 0.009(0.006-0.012) | ＜0.001 |
| FRS+cuCHG- WC | 0.602 (0.583-0.621) | 0.623 (0.584-0.663) | ＜0.001 | 0.242 (0.158-0.325) | ＜0.001 | 0.009 (0.006-0.012) | ＜0.001 |
| FRS+cuCHG-WHtR | 0.605 (0.586-0.623) | 0.628 (0.589-0.668) | ＜0.001 | 0.215 (0.131-0.300) | ＜0.001 | 0.010 (0.007-0.013) | ＜0.001 |
| FRS+cuCHG-VAI | 0.581 (0.562-0.600) | 0.611 (0.580-0.652) | 0.006 | 0.128 (0.048-0.208) | 0.002 | 0.008 (0.001-0.003) | 0.004 |
| FRS+cuCHG-CVAI | 0.604 (0.586-0.623) | 0.636 (0.596-0.675) | ＜0.001 | 0.262 (0.178-0.345) | ＜0.001 | 0.009 (0.006-0.011) | ＜0.001 |

CHG: cholesterol-high-density lipoprotein-glucose; CHG-ROD: the composite index of CHG and refined obesity derivatives; CVD: cardiovascular disease; FRS: Framingham Risk Score; cu, cumulative; AUC: area under the curve; CI: confidence interval; WC: waist circumference; WHtR: waist-to-height ratio; BMI: body mass index; VAI: visceral adiposity index; CVAI: CVD-associated adiposity index; NRI: net reclassification improvement, and IDI: integrated discrimination improvement.

**Supplementary Figure 2A. Subgroup analyses of baseline CHG-ROD in relation to CVD risk**

CHG: cholesterol-high-density lipoprotein-glucose, CHG-ROD: the composite index of CHG and refined obesity derivatives; CVD: cardiovascular disease; WC: waist circumference; WHtR: waist-to-height ratio; BMI: body mass index; VAI: visceral adiposity index, and CVAI: CVD-associated adiposity index.

**Supplementary Figure 2B. Subgroup analyses of cumulative CHG-ROD in relation to CVD risk**

CHG: cholesterol-high-density lipoprotein-glucose, CHG-ROD: the composite index of CHG and refined obesity derivatives; CVD: cardiovascular disease; WC: waist circumference; WHtR: waist-to-height ratio; BMI: body mass index; VAI: visceral adiposity index, and CVAI: CVD-associated adiposity index.

**Supplementary Table6. Subgroup analyses of trajectory CHG-ROD in relation to CVD risk**

| Variables | Cluster | HR (95%CI) | *P* | *P* for interaction |
| --- | --- | --- | --- | --- |
| CHG-BMI | | | | |
| Gender |  |  |  | 0.644 |
| Male | 1 | 1.00 (Reference) |  |  |
|  | 2 | 1.35 (1.07 ~ 1.69) |  |  |
| Female | 1 | 1.00 (Reference) |  |  |
|  | 2 | 1.28 (1.07 ~ 1.54) | 0.007 |  |
| Age, y |  |  |  | 0.275 |
| ＜65 | 1 | 1.00 (Reference) |  |  |
|  | 2 | 1.32 (1.12 ~ 1.55) | 0.001 |  |
| ≥65 | 1 | 1.00 (Reference) |  |  |
|  | 2 | 1.23 (0.93 ~ 1.63) | 0.152 |  |
| Drinking status |  |  |  | 0.791 |
| No | 1 | 1.00 (Reference) |  |  |
|  | 2 | 1.26 (1.07 ~ 1.49) | 0.007 |  |
| Yes | 1 | 1.00 (Reference) |  |  |
|  | 2 | 1.36 (1.05 ~ 1.77) | 0.022 |  |
| Smoking status |  |  |  | 0.389 |
| No | 1 | 1.00 (Reference) |  |  |
|  | 2 | 1.34 (1.14 ~ 1.57) | <.001 |  |
| Yes | 1 | 1.00 (Reference) |  |  |
|  | 2 | 1.19 (0.89 ~ 1.59) | 0.247 |  |
| Hypertension |  |  |  | 0.025 |
| No | 1 | 1.00 (Reference) |  |  |
|  | 2 | 1.56 (1.29 ~ 1.89) | <.001 |  |
| Yes | 1 | 1.00 (Reference) |  |  |
|  | 2 | 1.03 (0.84 ~ 1.27) | 0.749 |  |
| Diabetes |  |  |  | 0.883 |
| No | 1 | 1.00 (Reference) |  |  |
|  | 2 | 1.30 (1.12 ~ 1.52) | <.001 |  |
| Yes | 1 | 1.00 (Reference) |  |  |
|  | 2 | 1.18 (0.81 ~ 1.70) | 0.392 |  |
| Dyslipidemia |  |  |  | 0.569 |
| No | 1 | 1.00 (Reference) |  |  |
|  | 2 | 1.33 (1.14 ~ 1.54) | <.001 |  |
| Yes | 1 | 1.00 (Reference) |  |  |
|  | 2 | 1.10 (0.74 ~ 1.64) | 0.634 |  |
| CHG-WC | | | | |
| Gender |  |  |  | 0.951 |
| Male | 1 | 1.00 (Reference) |  |  |
|  | 2 | 1.42 (1.14 ~ 1.77) | 0.002 |  |
| Female | 1 | 1.00 (Reference) |  |  |
|  | 2 | 1.31 (1.09 ~ 1.57) | 0.004 |  |
| Age, y |  |  |  | 0.321 |
| ＜65 | 1 | 1.00 (Reference) |  |  |
|  | 2 | 1.38 (1.17 ~ 1.62) | <.001 |  |
| ≥65 | 1 | 1.00 (Reference) |  |  |
|  | 2 | 1.31 (1.00 ~ 1.71) | 0.051 |  |
| Drinking status |  |  |  | 0.624 |
| No | 1 | 1.00 (Reference) |  |  |
|  | 2 | 1.28 (1.08 ~ 1.51) | 0.005 |  |
| Yes | 1 | 1.00 (Reference) |  |  |
|  | 2 | 1.51 (1.17 ~ 1.94) | 0.001 |  |
| Smoking status |  |  |  | 0.671 |
| No | 1 | 1.00 (Reference) |  |  |
|  | 2 | 1.37 (1.16 ~ 1.61) | <.001 |  |
| Yes | 1 | 1.00 (Reference) |  |  |
|  | 2 | 1.33 (1.02 ~ 1.75) | 0.037 |  |
| Hypertension |  |  |  | 0.294 |
| No | 1 | 1.00 (Reference) |  |  |
|  | 2 | 1.48 (1.23 ~ 1.79) | <.001 |  |
| Yes | 1 | 1.00 (Reference) |  |  |
|  | 2 | 1.20 (0.98 ~ 1.48) | 0.082 |  |
| Diabetes |  |  |  | 0.429 |
| No | 1 | 1.00 (Reference) |  |  |
|  | 2 | 1.33 (1.15 ~ 1.55) | <.001 |  |
| Yes | 1 | 1.00 (Reference) |  |  |
|  | 2 | 1.45 (0.94 ~ 2.23) | 0.090 |  |
| Dyslipidemia |  |  |  | 0.832 |
| No | 1 | 1.00 (Reference) |  |  |
|  | 2 | 1.35 (1.16 ~ 1.57) | <.001 |  |
| Yes | 1 | 1.00 (Reference) |  |  |
|  | 2 | 1.37 (0.88 ~ 2.12) | 0.159 |  |
| CHG-WHtR | | | | |
| Gender |  |  |  | 0.436 |
| Male | 1 | 1.00 (Reference) |  |  |
|  | 2 | 1.32 (1.06 ~ 1.64) | 0.014 |  |
| Female | 1 | 1.00 (Reference) |  |  |
|  | 2 | 1.35 (1.11 ~ 1.63) | 0.002 |  |
| Age, y |  |  |  | 0.023 |
| ＜65 | 1 | 1.00 (Reference) |  |  |
|  | 2 | 1.42 (1.20 ~ 1.67) | <.001 |  |
| ≥65 | 1 | 1.00 (Reference) |  |  |
|  | 2 | 1.10 (0.84 ~ 1.46) | 0.485 |  |
| Drinking status |  |  |  | 0.904 |
| No | 1 | 1.00 (Reference) |  |  |
|  | 2 | 1.30 (1.10 ~ 1.55) | 0.003 |  |
| Yes | 1 | 1.00 (Reference) |  |  |
|  | 2 | 1.38 (1.07 ~ 1.77) | 0.014 |  |
| Smoking status |  |  |  | 0.331 |
| No | 1 | 1.00 (Reference) |  |  |
|  | 2 | 1.37 (1.16 ~ 1.62) | <.001 |  |
| Yes | 1 | 1.00 (Reference) |  |  |
|  | 2 | 1.21 (0.91 ~ 1.60) | 0.187 |  |
| Hypertension |  |  |  | 0.285 |
| No | 1 | 1.00 (Reference) |  |  |
|  | 2 | 1.45 (1.20 ~ 1.76) | <.001 |  |
| Yes | 1 | 1.00 (Reference) |  |  |
|  | 2 | 1.18 (0.96 ~ 1.46) | 0.120 |  |
| Diabetes |  |  |  | 0.229 |
| No | 1 | 1.00 (Reference) |  |  |
|  | 2 | 1.31 (1.12 ~ 1.52) | <.001 |  |
| Yes | 1 | 1.00 (Reference) |  |  |
|  | 2 | 1.48 (0.97 ~ 2.28) | 0.071 |  |
| Dyslipidemia |  |  |  | 0.876 |
| No | 1 | 1.00 (Reference) |  |  |
|  | 2 | 1.34 (1.15 ~ 1.56) | <.001 |  |
| Yes | 1 | 1.00 (Reference) |  |  |
|  | 2 | 1.22 (0.81 ~ 1.83) | 0.337 |  |
| CHG-VAI | | | | |
| Gender |  |  |  | 0.968 |
| Male | 1 | 1.00 (Reference) |  |  |
|  | 2 | 1.34 (0.88 ~ 2.05) | 0.175 |  |
| Female | 1 | 1.00 (Reference) |  |  |
|  | 2 | 1.13 (0.88 ~ 1.43) | 0.337 |  |
| Age, y |  |  |  | 0.801 |
| ＜65 | 1 | 1.00 (Reference) |  |  |
|  | 2 | 1.18 (0.93 ~ 1.50) | 0.162 |  |
| ≥65 | 1 | 1.00 (Reference) |  |  |
|  | 2 | 1.27 (0.80 ~ 2.03) | 0.310 |  |
| Drinking status |  |  |  | 0.396 |
| No | 1 | 1.00 (Reference) |  |  |
|  | 2 | 1.22 (0.97 ~ 1.54) | 0.091 |  |
| Yes | 1 | 1.00 (Reference) |  |  |
|  | 2 | 1.09 (0.67 ~ 1.77) | 0.733 |  |
| Smoking status |  |  |  | 0.327 |
| No | 1 | 1.00 (Reference) |  |  |
|  | 2 | 1.13 (0.89 ~ 1.42) | 0.320 |  |
| Yes | 1 | 1.00 (Reference) |  |  |
|  | 2 | 1.51 (0.94 ~ 2.42) | 0.086 |  |
| Hypertension |  |  |  | 0.749 |
| No | 1 | 1.00 (Reference) |  |  |
|  | 2 | 1.17 (0.84 ~ 1.63) | 0.348 |  |
| Yes | 1 | 1.00 (Reference) |  |  |
|  | 2 | 1.19 (0.91 ~ 1.56) | 0.207 |  |
| Diabetes |  |  |  | 0.205 |
| No | 1 | 1.00 (Reference) |  |  |
|  | 2 | 1.11 (0.85 ~ 1.45) | 0.457 |  |
| Yes | 1 | 1.00 (Reference) |  |  |
|  | 2 | 1.31 (0.92 ~ 1.86) | 0.134 |  |
| Dyslipidemia |  |  |  | 0.996 |
| No | 1 | 1.00 (Reference) |  |  |
|  | 2 | 1.17 (0.75 ~ 1.82) | 0.486 |  |
| Yes | 1 | 1.00 (Reference) |  |  |
|  | 2 | 1.21 (0.95 ~ 1.53) | 0.120 |  |
| CHG-CVAI | | | | |
| Gender |  |  |  | 0.580 |
| Male | 1 | 1.00 (Reference) |  |  |
|  | 2 | 1.34 (1.08 ~ 1.67) | 0.007 |  |
| Female | 1 | 1.00 (Reference) |  |  |
|  | 2 | 1.39 (1.16 ~ 1.68) | <.001 |  |
| Age, y |  |  |  | 0.113 |
| ＜65 | 1 | 1.00 (Reference) |  |  |
|  | 2 | 1.41 (1.20 ~ 1.66) | <.001 |  |
| ≥65 | 1 | 1.00 (Reference) |  |  |
|  | 2 | 1.24 (0.94 ~ 1.64) | 0.136 |  |
| Drinking status |  |  |  | 0.691 |
| No | 1 | 1.00 (Reference) |  |  |
|  | 2 | 1.31 (1.11 ~ 1.55) | 0.002 |  |
| Yes | 1 | 1.00 (Reference) |  |  |
|  | 2 | 1.47 (1.15 ~ 1.89) | 0.002 |  |
| Smoking status |  |  |  | 0.426 |
| No | 1 | 1.00 (Reference) |  |  |
|  | 2 | 1.39 (1.18 ~ 1.64) | <.001 |  |
| Yes | 1 | 1.00 (Reference) |  |  |
|  | 2 | 1.27 (0.97 ~ 1.67) | 0.080 |  |
| Hypertension |  |  |  | 0.218 |
| No | 1 | 1.00 (Reference) |  |  |
|  | 2 | 1.48 (1.22 ~ 1.78) | <.001 |  |
| Yes | 1 | 1.00 (Reference) |  |  |
|  | 2 | 1.23 (1.00 ~ 1.51) | 0.052 |  |
| Diabetes |  |  |  | 0.350 |
| No | 1 | 1.00 (Reference) |  |  |
|  | 2 | 1.33 (1.14 ~ 1.54) | <.001 |  |
| Yes | 1 | 1.00 (Reference) |  |  |
|  | 2 | 1.51 (1.02 ~ 2.22) | 0.040 |  |
| Dyslipidemia |  |  |  | 0.971 |
| No | 1 | 1.00 (Reference) |  |  |
|  | 2 | 1.35 (1.17 ~ 1.57) | <.001 |  |
| Yes | 1 | 1.00 (Reference) |  |  |
|  | 2 | 1.37 (0.91 ~ 2.07) | 0.133 |  |

CHG: cholesterol-high-density lipoprotein-glucose; CHG-ROD: the composite index of CHG and refined obesity derivatives; CVD: cardiovascular disease; HR: hazard ratio; CI: confidence interval; WC: waist circumference; WHtR: waist-to-height ratio; BMI: body mass index; VAI: visceral adiposity index, and CVAI: CVD-associated adiposity index.

**Supplementary Table7. Association of CHG-ROD with CVD Risk After Excluding Participants with Recent CVD**

| Variables | OR (95%CI) | *P* |
| --- | --- | --- |
| CHG-BMI (Per SD increase) | 1.19 (1.04 ~ 1.36) | 0.012 |
| CHG-WC (Per SD increase) | 1.17 (1.03 ~ 1.35) | 0.020 |
| CHG-WHtR (Per SD increase) | 1.16 (1.01 ~ 1.33) | 0.037 |
| CHG-VAI (Per SD increase) | 0.99 (0.87 ~ 1.12) | 0.860 |
| CHG-CVAI (Per SD increase) | 1.20 (1.05 ~ 1.37) | 0.006 |
| cuCHG-BMI (Per SD increase) | 1.18 (1.04 ~ 1.35) | 0.013 |
| cuCHG-WC (Per SD increase) | 1.17 (1.02 ~ 1.33) | 0.024 |
| cuCHG-WHtR (Per SD increase) | 1.15 (1.01 ~ 1.32) | 0.042 |
| cuCHG-VAI (Per SD increase) | 1.00 (0.89 ~ 1.13) | 0.969 |
| cuCHG-CVAI (Per SD increase) | 1.20 (1.05 ~ 1.37) | 0.007 |
| CHG-BMI Cluster |  |  |
| 1 | 1.00 (Reference) |  |
| 2 | 1.31 (1.01 ~ 1.71) | 0.044 |
| CHG-WC Cluster |  |  |
| 1 | 1.00 (Reference) |  |
| 2 | 1.33 (1.03 ~ 1.73) | 0.031 |
| CHG-WHtR Cluster |  |  |
| 1 | 1.00 (Reference) |  |
| 2 | 1.38 (1.06 ~ 1.80) | 0.019 |
| CHG-VAI Cluster |  |  |
| 1 | 1.00 (Reference) |  |
| 2 | 1.16 (0.77 ~ 1.74) | 0.488 |
| CHG-CVAI Cluster |  |  |
| 1 | 1.00 (Reference) |  |
| 2 | 1.43 (1.10 ~ 1.85) | 0.007 |
| CHG-BMI quantile |  |  |
| 1 |  |  |
| 2 | 1.12 (0.77 ~ 1.63) | 0.567 |
| 3 | 1.23 (0.85 ~ 1.80) | 0.274 |
| 4 | 1.65 (1.13 ~ 2.42) | 0.010 |
| P for trend |  | 0.006 |
| CHG-WC quantile |  |  |
| 1 | 1.00 (Reference) |  |
| 2 | 1.01 (0.68 ~ 1.49) | 0.965 |
| 3 | 1.39 (0.96 ~ 2.00) | 0.081 |
| 4 | 1.72 (1.18 ~ 2.49) | 0.004 |
| P for trend |  | 0.001 |
| CHG-WHtR quantile |  |  |
| 1 | 1.00 (Reference) |  |
| 2 | 1.02 (0.70 ~ 1.50) | 0.912 |
| 3 | 1.35 (0.93 ~ 1.95) | 0.114 |
| 4 | 1.59 (1.08 ~ 2.33) | 0.018 |
| P for trend |  | 0.007 |
| CHG-VAI quantile |  |  |
| 1 | 1.00 (Reference) |  |
| 2 | 1.36 (0.94 ~ 1.96) | 0.098 |
| 3 | 1.31 (0.91 ~ 1.90) | 0.150 |
| 4 | 1.27 (0.86 ~ 1.86) | 0.227 |
| P for trend |  | 0.582 |
| CHG-CVAI quantile |  |  |
| 1 | 1.00 (Reference) |  |
| 2 | 0.93 (0.63 ~ 1.37) | 0.720 |
| 3 | 1.28 (0.89 ~ 1.85) | 0.182 |
| 4 | 1.51 (1.04 ~ 2.19) | 0.030 |
| P for trend |  | 0.009 |
| cuCHG-BMI quantile |  |  |
| 1 |  |  |
| 2 | 0.99 (0.68 ~ 1.44) | 0.951 |
| 3 | 1.11 (0.77 ~ 1.61) | 0.577 |
| 4 | 1.54 (1.06 ~ 2.24) | 0.024 |
| P for trend |  | 0.012 |
| cuCHG-WC quantile |  |  |
| 1 |  |  |
| 2 | 0.91 (0.62 ~ 1.33) | 0.627 |
| 3 | 1.26 (0.88 ~ 1.80) | 0.211 |
| 4 | 1.46 (1.01 ~ 2.10) | 0.042 |
| P for trend |  | 0.014 |
| cuCHG-WHtR quantile |  |  |
| 1 | 1.00 (Reference) |  |
| 2 | 0.94 (0.64 ~ 1.38) | 0.761 |
| 3 | 1.39 (0.97 ~ 1.99) | 0.070 |
| 4 | 1.61 (1.11 ~ 2.32) | 0.011 |
| P for trend |  | 0.002 |
| cuCHG-VAI quantile |  |  |
| 1 | 1.00 (Reference) |  |
| 2 | 0.98 (0.68 ~ 1.42) | 0.918 |
| 3 | 1.24 (0.86 ~ 1.77) | 0.244 |
| 4 | 1.12 (0.76 ~ 1.64) | 0.575 |
| P for trend |  | 0.532 |
| cuCHG-CVAI quantile |  |  |
| 1 | 1.00 (Reference) |  |
| 2 | 1.17 (0.81 ~ 1.70) | 0.408 |
| 3 | 1.40 (0.97 ~ 2.02) | 0.076 |
| 4 | 1.84 (1.25 ~ 2.71) | 0.002 |
| P for trend |  | 0.004 |

CHG: cholesterol-high-density lipoprotein-glucose; CHG-ROD: the composite index of CHG and refined obesity derivatives; CVD: cardiovascular disease; OR: odds ratio; CI: confidence interval, cu: cumulative; WC: waist circumference; WHtR: waist-to-height ratio; BMI: body mass index; VAI: visceral adiposity index, and CVAI: CVD-associated adiposity index.
